# Comparative Study between Full-Endoscopic Discectomy and Microendoscopic Discectomy for the Treatment of Lumbar Disc Herniation

**DOI:** 10.3390/medicina56120710

**Published:** 2020-12-18

**Authors:** Muneyoshi Fujita, Tomoaki Kitagawa, Masahiro Hirahata, Takahiro Inui, Hirotaka Kawano, Hiroki Iwai, Hirohiko Inanami, Hisashi Koga

**Affiliations:** 1 Department of Orthopaedics, Iwai Orthopaedic Medical Hospital, 8-17-2 Minamikoiwa Edogawa-ku, Tokyo 133-0056, Japan; rocky5477@gmail.com (H.I.); inanamihiro@gmail.com (H.I.); hkoga0808@gmail.com (H.K.); 2 Department of Orthopaedic Surgery, Teikyo University School of Medicine, 2-11-1 Kaga, Itabashi-ku, Tokyo 173-8606, Japan; tomoakikitagawa1012@gmail.com (T.K.); pleasure-masa@cyber.ocn.ne.jp (M.H.); johnclerk2003@hotmail.com (T.I.); hkawano-tky@med.teikyo-u.ac.jp (H.K.); 3 Department of Neurosurgery, Iwai FESS Clinic, 8-18-4 Minamikoiwa Edogawa-ku, Tokyo 133-0056, Japan; 4 Department of Orthopaedic Surgery, Inanami Spine and Joint Hospital, 3-17-5 Higashishinagawa Shinagawa-ku, Tokyo 140-0002, Japan

**Keywords:** lumbar disc herniation, full-endoscopic spine surgery, discectomy, minimally invasive, radiculopathy, low back pain

## Abstract

*Background and objectives*: Lumbar disc herniation (LDH) is a common disease in the meridian of life. Although surgical discectomy is commonly used to treat LDH, there are several different strategies. We compared the outcomes of uniportal full-endoscopic discectomy (FED) with those of microendoscopic discectomy (MED) in treating LDH. *Materials and Methods*: FED was performed using a 4.1-mm working channel endoscope, and MED was performed using a 16-mm diameter tubular retractor and endoscope. Data of patients with LDH treated with FED (*n* = 39) or MED (*n* = 27) by the single surgeon were retrospectively reviewed. Patient background information and operative data were collected. Pre- and postoperative low back and leg pain were evaluated using the numerical rating scale (NRS) score. Pre- and postoperative disc height index (DHI) values were calculated from plain radiographs, and the disc height loss was evaluated using the ratio (DHI ratio); *Results*: The median (interquartile range (IQR) Q25–75) operation times for FED and MED were 42 (33–61) and 43 (33–50) minutes, respectively. The median (IQR Q25–75) pre- and postoperative NRS scores for low back pain were 5 (2–7) and 1 (0–4), respectively, for FED and 6 (3–8) and 1 (0–2), respectively, for MED. The median (IQR Q25–75) pre- and postoperative NRS scores for leg pain were 7 (5–8) and 0 (0–2), respectively, for FED and 6 (5–8) and 0 (0–2), respectively, for MED. These data were not different between the FED and MED groups. The median (IQR Q25–75) DHI ratios of FED and MED were 0.94 (0.89–1.03) and 0.90 (0.79–0.95), respectively. The DHI ratio was significantly higher (*p* < 0.05) in the FED group than in the MED group, and there was less blood loss; *Conclusions*: The pain-relieving effect of FED in treating LDH was almost identical to that of MED. However, FED was superior to MED in preventing disc height loss, which is one of the indicators of postoperative disc degeneration.

## 1. Introduction

Microendoscopic discectomy (MED) is an intramuscular paramedian approach using a 16-mm diameter tubular retractor and endoscope. MED was first described by Foley et al. in the United States. [1,2]. However, it has been further developed mainly in Japan because of its minimally invasive nature. More than 10,000 patients with lumbar disc herniation (LDH) undergo MED annually in Japan.

We previously demonstrated good surgical results with MED regardless of the presence of spondylolysis in patients with sciatica with concomitant LDH [3]. Other investigators also reported the superiority of MED to conventional open surgery for treating LDH [4,5]. The advantages of its minimal invasiveness (less trauma and less bleeding) and short hospital stay were emphasized in these studies. Phan et al. performed a meta-analysis comparing the outcomes of full-endoscopic discectomy (FED), MED, and open discectomy (OD) (23 studies: 421 patients in the FED group, 6914 patients in the MED group, and 21,152 patients in the OD group) [6]. They concluded that FED and MED appear to be safe and efficacious alternatives to traditional approaches. However, these results require further investigations [6].

In contrast, uniportal FED is considered to be more minimally invasiveness than MED because of the small skin incision and saline irrigation required during the surgery. Most recently, one- and two-year results of a prospective randomized controlled studies of FED and MED were reported by Chen et al. [7,8]. Not only was the superiority of FED on clinical outcomes and safety not shown in these studies, but also FED had inferior results for median disc herniation. Although the surgeries were performed by a number of skilled surgeons (qualified in minimally invasive spine surgery (MISS), with >3 years of experience, and 200 MISS procedures performed) in these studies, we have to consider the effect of different surgical skills because the outcomes of FED highly depend on surgical skills [9].

Therefore, this study aimed to compare the outcomes of FED with those of MED performed by a single skilled surgeon and examine the long-term effects of the procedures on disc degeneration, which have not been extensively examined in previous studies.

## 2. Materials and Methods

Study design: Retrospective case-control study.

### 2.1. Patient Selection

**Figure 1:**
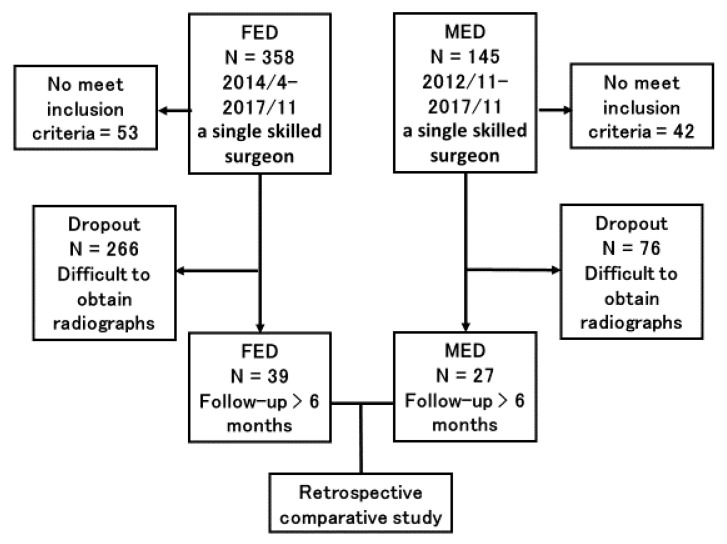
Study design. FED = full-endoscopic discectomy; MED = microendoscopic discectomy.

FED or MED was performed in 503 consecutive patients with LDH between November 2012 and November 2017 by a single skilled surgeon (H.K. who has more than 1000 surgical experiences for FED and MED). We explained the merits and the demerits of both procedures to patients and the patients determined MED or FED. There was no bias of size and types of LDH. All patients had LDH at only one vertebral level with radiculopathy; resistant to medical treatment, epidural steroids, and/or nerve block. We only included patients who underwent follow-up plain radiography 6 months after the surgery. We excluded patients with recurrent LDH and those who had a past history of other spinal surgeries. We also excluded patients with intra- and extraforaminal LDH because of the different operative route (Figure 1). Background information of the patients, including age, sex, height, body mass index (BMI), smoking history, and the operated vertebral level, were obtained from medical records (Table 1). Operation time, hospital stay, and complications related to the surgery were also collected.

### 2.2. Surgical Procedures

The patients were carefully logrolled into the prone position. Surgery was performed under general anesthesia combined with motor evoked potential monitoring. During the surgery, a fluoroscope was placed across the center of the operative table to ensure appropriate timing.

For MED, an 18-mm skin incision was made 10-mm lateral to the midline and then a METRx endoscopic system (Medtronic Sofamor Danek, Memphis, TN, USA) was inserted. The basic operative procedure has been previously described [3,10]. Especially for enlargement of bone window, laminectomy was performed mainly using a chisel (width: 4 mm), and thereby a drain was placed. For FED, an 8-mm skin incision was made and then a 4.1-mm working channel endoscope (RIWOspine GmbH, Knittlingen, Germany) was inserted. Two different FED approaches were performed in this study, namely, an interlaminar approach (ILA) and transforaminal approach (TFA). Only outside-in technique was performed for TFA [11]. The selective fragmentectomy was performed for both ILA and TFA and normal nucleus pulposus was preserved [12]. The detailed operative procedures have been previously described [13,14,15].

### 2.3. Evaluation of Pain

Pre- and postoperative pain in the low back and legs were evaluated using the numerical rating scale (NRS) score. The postoperative NRS score was obtained at discharge from the hospital.

### 2.4. Evaluation of LDH Size Removed Disc Weight

LDH size was calculated by two methods. The weight of removed disc material was calculated immediately after the removal and recorded on the medical record. The occupancy ratio of the spinal canal by the protruded nucleus pulposus (NP) was measured on axial T2-weighted MRI. The areas of protruded NP and the corresponding spinal canal were calculated using an image measurement software. The area of the spinal canal was defined as the region enclosed by the original dorsal surface of the annulus fibrosus and the ventral edge of the ligamentum flavum. The area of the protruded NP was defined as the region enclosed by the original dorsal surface of the annulus fibrosus and the dorsal surface of the protruded NP. The occupancy ratio was calculated using the following formula: occupancy ratio of the spinal canal = the area of the protruded NP (blue line area)/the area of the spinal canal (red line) × 100 (%) [16].

### 2.5. Statistical Analysis

Categorical variables are reported as frequencies and percentages. Continuous variables are shown as median and interquartile range (IQR Q25–75). Demographic data and outcomes were compared between FED and MED groups using Mann–Whitney U test for continuous variables and chi-square test for categorical variables. All analyses were performed using STATA (version 16.0; Stata Corp LLC, College Station, TX, USA). A two-sided *p*-value < 0.05 was considered statistically significant.

### 2.6. Evaluation of Disc Height

**Figure 2:**
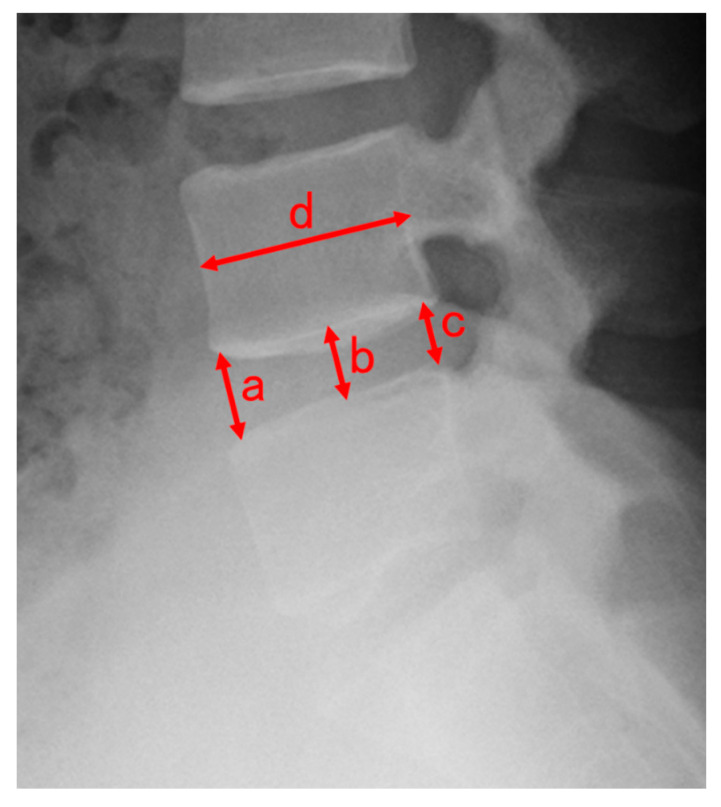
Radiographic measurements of the lumbar disc height. (**a**): anterior disc height, (**b**): middle disc height, (**c**): posterior disc height, (**d**): sagittal diameter of the overlying vertebral body. Disc height = (a + b + c)/3 (mm). Disc height index = disc height/d.

Pre- and postoperative (at least 6 months after the surgery) plain radiographs were used to calculate the DHI ratio. Plain radiographs of the lumbar spine were taken in the standing upright and neutral positions. The disc height was calculated as the mean of the anterior, middle, and posterior disc heights: disc height = (a + b + c)/3 (mm). The sagittal diameter (d) of the vertebral body from the anterior to posterior margin was measured at the mid-vertebral level. The disc height index (DHI) was calculated using the following formula: disc height index = mean disc height/d [17,18]. The DHI ratio was calculated as postoperative DHI/preoperative DHI (Figure 2).

## 3. Results

Demographic data are summarized in Table 1. This case series consists of 39 patients in the FED group (25 males, 14 females) and 27 patients in the MED group (17 males, 10 females). The FED group comprised 24 cases of ILA (61.5%) and 15 cases of TFA outside-in technique (38.5%). The median (IQR Q25–75) ages at surgery were 46 (39–53) and 44 (28–55) years in the FED and MED groups, respectively. The most commonly affected vertebral levels were L5/S1 and L4/5 in the FED (56.4%) and MED (63.0%) groups, respectively. There were no significant differences in patient background between both groups except for the affected vertebral level.

There was no significant difference in the median (IQR Q25–75) operation time between the FED group 42 (33–61) min and MED group 43 (33–50) min. There was a significant difference in the median (IQR Q25–75) hospital stay between the FED group 2 (1–3) days and MED group 5 (4–5) days (*p* < 0.05) (Table 2). There was also a significant difference in the median (IQR Q25–75) blood loss between the FED group (not determined) and MED group 60 (50–90) ml (*p* < 0.05). Intraoperative complications, such as dural tear and nerve injuries, were not observed in any patients in either the FED or MED group. No other postoperative complications, such as surgical site infection or postoperative hematoma, were observed. There was no significant difference in the weight of removed disc material and the occupancy ratio measured on axial T2-weighted MRI.

The median (IQR Q25–75) pre- and postoperative NRS scores for low back pain were 5 (2–7) and 1 (0–4), respectively, in the FED group and 6 (3–8) and 1 (0–2), respectively, in the MED group. The median (IQR Q25–75) pre- and postoperative NRS scores for leg pain were 7 (5–8) and 0 (0–2), respectively, in the FED group and 6 (5–8) and 0 (0–2), respectively, in the MED group. These data were not different between the FED and MED groups. The median (IQR Q25–75) DHI ratios in the FED and MED groups were 0.94 (0.89–1.03) and 0.90 (0.79–0.95), respectively. The DHI ratio was significantly higher (*p* < 0.05) in the FED group, which also had a shorter hospital stay and less blood loss (Table 2).

## 4. Discussion

Uniportal FED has several different operative approaches, such as TFA, ILA, posterolateral, and translaminar. Each approach has different indications depending on the location and types of LDH. For example, caudally sequestrate L5/S1 LDH should be treated using ILA. In contrast, most intracanal LDH can be treated using an intramuscular paramedian MED approach regardless of the extent of laminectomy. We thus targeted intracanal LDH that was treated via an intramuscular paramedian MED approach or TFA/ILA FED approach. We considered that extracanal LDH (foraminal and extraforaminal) should be analyzed differently and thus excluded this from our study because of the completely different operative approaches.

Several previous meta-analyses comparing the outcomes of FED with those of MED have already been reported. Among such studies, Zhao et al. indicated that FED-TFA has a number of advantages owing to its minimally invasive nature, but subsequent recurrence and revision rates are higher than those of MED (12 studies: 1048 patients in the FED group and 1352 patients in the MED group). Zhao et al. concluded that MED should not be completely replaced by TFA-FED [19]. Although Xu et al. performed a similar meta-analysis (nine studies: 468 patients in the FED group and 516 patients in the MED group), no differences in leg pain were found, although lower back pain in the FED group was lesser than that in the MED group 24 months after surgery [20]. The efficacy of FED for treating low back pain was also reported in another meta-analysis by Yu et al. [21]. Xu et al. also reported that there were no significant differences in complication, recurrence, or reoperation rates between FED and MED. Chen et al. performed a meta-analysis of specified complications (17 studies: 258 patients in the FED group, 288 patients in the MED group, and 1018 patients in the OD/microdiscectomy [OD/MD] group) and found a lower risk of complications in the FED group than in the OD/MD group. There was no significant difference between the MED and OD/MD groups, but the FED and MED groups were not directly compared [22,23]. In their meta-analysis, Feng et al. also reported the superiority of FED with respect to complications [24]. Shi et al. performed a maximal meta-analysis (18 studies: 1093 patients in the FED group and 1068 patients in the MED group), but found no differences with respect to operative outcomes, Oswestry Disability Index (ODI) and visual analog scale (VAS), duration of operation, total complication rate, or recurrence rate [25]. Shi et al. also reported that FED led to less trauma and bleeding, shorter hospital stays, and better outcomes with respect to low back pain. Even after many meta-analyses, there remain a number of controversies.

Based on our analysis, operative outcomes as indicated by NRS scores for low back pain and leg pain were not significantly different between the FED and MED groups. We observed no complications in either groups. Therefore, both operative procedures appear to be appropriate and successful modern treatments for intracanal LDH. However, the hospital stay was significantly shorter and there was significantly less blood loss in the FED group than in the MED group, similar to some previous studies [26,27]. In addition to these differences, we found that the DHI ratio was significant higher (*p* < 0.05) in the FED group than in the MED group. Because disc height loss is one of the indicators of postoperative disc degeneration [28], the prevention of disc height loss seems to be an advantage that has not been identified in previous studies. To integrally decide the superiority of a surgical procedure from our study, FED was the more minimally invasive and more protective procedure for intervertebral disc. We used plain radiographs to assess disc degeneration according to traditional methods, but patients relieved from pain did not want to be exposed to radiation. We thus have to use alternative evaluation methods, such as magnetic resonance imaging, to reduce radiation exposure. Further extensive studies are required to prove the superiority of FED with respect to the prevention of postoperative disc degeneration.

### Limitation of This Study

This study had some limitations. First, the study was a retrospective analysis. Second, the sample size was relatively small to obtain a definitive conclusion. Third, the number of dropouts was extremely high because of the refusal to undergo further radiation exposure. Fourth, additional follow-up parameters, such as ODI or MOS 36-Item Short-Form Health Survey (SF-36), should be examined.

## 5. Conclusions

Preliminary results over a short follow-up period showed that the operative outcomes of FED were similar to those of MED in the treatment of intracanal LDH. FED is less invasive than MED, thus FED has less blood loss. Disc height loss, which is one of the indicators of postoperative disc degeneration, was significantly prevented by FED.

## Figures and Tables

**Table 1:** Demographic data.

Characteristic	FED (*N* = 39)	MED (*N* = 27)	*p* Value
Age (years), median (IQR Q25–75)	46 (39–53)	44 (28–55)	0.25
Sex male, *n* (%)	25 (64.1%)	17 (63.0%)	0.92
Height (cm), median (IQR Q25–75)	168 (160–177)	164 (161–170)	0.44
BMI (kg/m^2^), median (IQR Q25–75)	24.56 (21.48–26.23)	23.80 (20.08–26.77)	0.62
Smoking history, *n* (%)	9 (23.1%)	8 (29.6%)	0.55
Surgical level, number, *n* (%)			<0.05
L1/2	2 (5.1%)	0	
L2/3	3 (7.7%)	0	
L3/4	5 (12.8%)	0	
L4/5	7 (17.9%)	17 (63.0%)	
L5/S1	22 (56.4%)	10 (37.0%)	
Follow-up Period (months), median (IQR Q25–75)	20 (12–31)	17 (12–36)	0.98

FED = full-endoscopic discectomy; MED = microendoscopic discectomy; SD = standard deviation; BMI = body mass index.

**Table 2:** Operative outcomes of 66 patients.

Parameter	FED (*N* = 39)	MED (*N* = 27)	*p* Value
NRS scores for Low Back, median (IQR Q25–75)			
Preoperative	5 (2–7)	6 (3–8)	0.6
At Discharge	1 (0–4)	1 (0–2)	0.47
NRS scores for Leg, median (IQR Q25–75)			
Preoperative	7 (5–8)	6 (5–8)	0.91
At Discharge	0 (0–2)	0 (0–2)	0.46
Preoperative DHI, median (IQR Q25–75)	0.23 (0.19–0.29)	0.24 (0.22–0.27)	0.43
Postoperative DHI, median (IQR Q25–75)	0.22 (0.18–0.27)	0.22 (0.20–0.24)	0.69
DHI ratio, median (IQR Q25–75)	0.94 (0.89–1.03)	0.90 (0.79–0.95)	<0.05
Operation Time (minutes), median (IQR Q25–75)	42.0 (33.0–61.0)	43.0 (33.0–50.0)	0.79
Blood Loss (ml), median (IQR Q25–75)	<50	60 (50–90)	<0.05
Postoperative Hospital Stay (days), median (IQR Q25–75)	2 (1–3)	5 (4–5)	<0.05
Weight of LDH (g), median (IQR Q25–75)	0.60 (0.50–1.10)	0.80 (0.40–1.90)	0.17
maximum occupancy ratio of LDH on axial MRI (%), median (IQR Q25–75)	26.0 (17.3–36.7)	29.1 (18.6–37.0)	0.75

FED = full-endoscopic discectomy; MED = microendoscopic discectomy; NRS = numerical rating scale; IQR = interquartile range; DHI = disc height index; LDH = lumbar disc herniation; MRI = magnetic resonance imaging.
